# Letter from the Editor in Chief

**DOI:** 10.19102/icrm.2024.15056

**Published:** 2024-05-15

**Authors:** Moussa Mansour


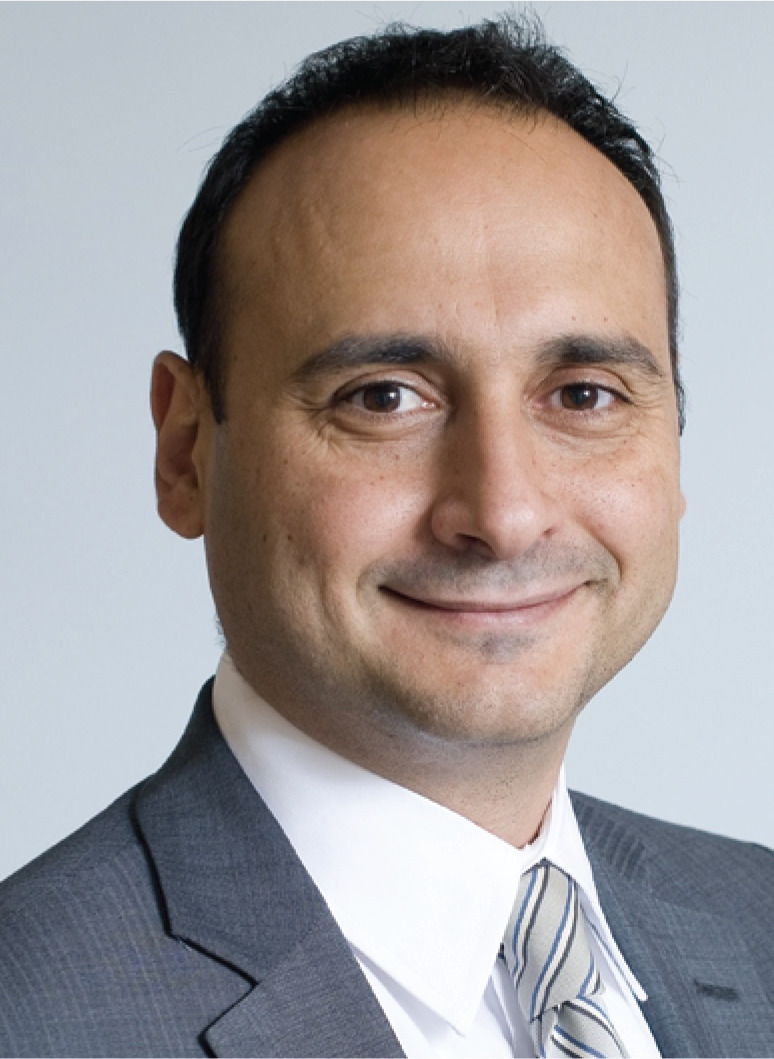


Dear readers,

In my monthly letter in June 2023, I discussed the emerging field of left bundle branch area pacing (LBBAP).^1^ In the year since, this field has continued to grow rapidly, gathering significant interest from the cardiac electrophysiology community. One article on this topic I would like to highlight is published in this issue of *The Journal of Innovations in Cardiac Rhythm Management*. The article, titled “Clinical and Electrophysiological Outcomes of Left Bundle Area Pacing Compared to Biventricular Pacing: An Updated Meta-analysis,” by Lakshman et al., reports the result of a meta-analysis comparing the structural, electrophysiological, clinical, and procedural outcomes of LBBAP and biventricular pacing (BVP).^2^ Nine studies, two of which were randomized, were included in the analysis, which revealed that LBBAP, compared to BVP, was associated with greater reductions in the left ventricular volume, QRS duration, New York Heart Association functional class, heart failure hospitalizations, and procedural fluoroscopic time.

To date, a significant number of studies have been conducted to confirm the benefit of LBBAP in different patient populations and compare it with BVP or right ventricular (RV) pacing. Most of these studies have been observational, except for a small number of randomized studies enrolling only small numbers of patients. As a result, LBBAP has not yet been established as the recommended approach for pacing in patients with and without heart failure.

A number of ongoing large multicenter clinical trials are currently underway to confirm the benefits of LBBAP seen in smaller studies. One such study is the Left vs. Left Randomized Clinical Trial (ClinicalTrials.gov identifier: NCT05650658), a randomized, multicenter clinical trial comparing the effectiveness of His or LBBAP versus BVP in patients with heart failure due to left ventricular systolic dysfunction (LVEF ≤ 50%) and either a wide QRS (≥130 ms) or with/anticipated to have >40% pacing. The primary study endpoint of this trial is a composite of all-cause mortality and hospitalization for heart failure. The study aims to enroll 2136 participants with an estimated date of completion in June 2029. Another important study is the Conduction System Pacing with Left Bundle Branch Pacing as Compared to Standard Right Ventricular Pacing study (ClinicalTrials.gov identifier: NCT05015660); this study is also a multicenter randomized trial comparing LBBAP and RV pacing in patients with an ejection fraction of >35%, an indication for pacing, and high-degree atrioventricular block where the degree of anticipated RV pacing is >90%. The study has three primary outcome measures: time to cardiovascular death, time to first heart failure event, and worsening LV end-systolic volume index at 2 years. The study aims to enroll 1300 participants, with an estimated date of completion in July 2027.

Considering all of the above, I believe that LBBAP will continue to gain momentum as a beneficial mode of pacing across a wide spectrum of patients who require pacing. Whether or not it becomes the primary mode of pacing will depend upon the findings of multicenter clinical randomized studies such as the ones described above.


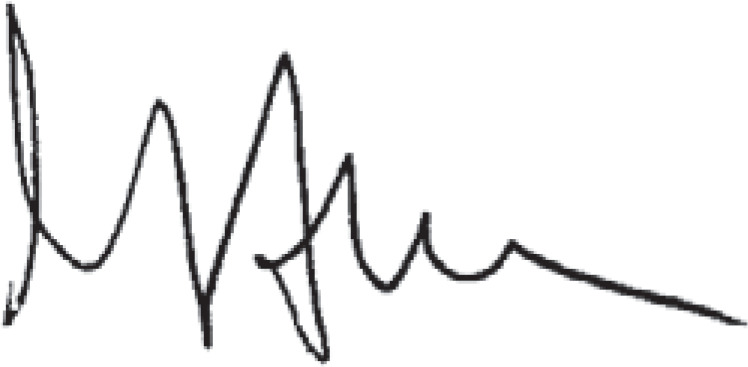


Best regards,

Moussa Mansour, md, fhrs, facc

Editor in Chief

*The Journal of Innovations in Cardiac Rhythm Management*

MMansour@InnovationsInCRM.com

Director, Atrial Fibrillation Program

Jeremy Ruskin and Dan Starks Endowed Chair in Cardiology

Massachusetts General Hospital

Boston, MA 02114
